# Glycemic response to meals with a high glycemic index differs between morning and evening: a randomized cross-over controlled trial among students with early or late chronotype

**DOI:** 10.1007/s00394-024-03372-4

**Published:** 2024-04-12

**Authors:** Bianca Stutz, Bettina Krueger, Janina Goletzke, Nicole Jankovic, Ute Alexy, Christian Herder, Jutta Dierkes, Gabriele Berg-Beckhoff, Rasmus Jakobsmeyer, Claus Reinsberger, Anette E. Buyken

**Affiliations:** 1 Faculty of Sciences, Institute of Nutrition, Consumption and Health, Paderborn University, Paderborn, Germany; 2 Nutritional Epidemiology, Department of Nutrition and Food Sciences, Rheinische Friedrich-Wilhelms-University Bonn, DONALD Study Centre, Dortmund, Germany; 3 Institute for Clinical Diabetology, German Diabetes Center (DDZ), Leibniz Center for Diabetes Research at Heinrich Heine University Düsseldorf, Düsseldorf, Germany; 4 German Center for Diabetes Research (DZD), Partner Düsseldorf, Munich-Neuherberg, Germany; 5 Department of Endocrinology and Diabetology, Medical Faculty and University Hospital Düsseldorf, Heinrich Heine University Düsseldorf, Düsseldorf, Germany; 6 Department of Clinical Medicine Center, University of Bergen, Bergen, Norway; 7 The Faculty of Health Sciences, Department of Public Health, University of Southern Denmark, Esbjerg, Denmark; 8 Faculty of Sciences, Institute of Sports Medicine, Paderborn University, Paderborn, Germany

**Keywords:** Chronotype, Circadian misalignment, Glucose homeostasis, Glycemic index, Meal time

## Abstract

**Purpose:**

Glycemic response to the same meal depends on daytime and alignment of consumption with the inner clock, which has not been examined by individual chronotype yet. This study examined whether the 2-h postprandial and 24-h glycemic response to a meal with high glycemic index (GI) differ when consumed early or late in the day among students with early or late chronotype.

**Methods:**

From a screening of 327 students aged 18–25 years, those with early (n = 22) or late (n = 23) chronotype participated in a 7-day randomized controlled cross-over intervention study. After a 3-day observational phase, standardized meals were provided on run-in/washout (days 4 and 6) and intervention (days 5 and 7), on which participants received a high GI meal (GI = 72) in the morning (7 a.m.) or in the evening (8 p.m.). All other meals had a medium GI. Continuous glucose monitoring was used to measure 2-h postprandial and 24-h glycemic responses and their variability.

**Results:**

Among students with early chronotype 2-h postprandial glucose responses to the high GI meal were higher in the evening than in the morning (iAUC: 234 (± 92) vs. 195 (± 91) (mmol/L) × min, p = 0.042). Likewise, mean and lowest 2-h postprandial glucose values were higher when the high GI meal was consumed in the evening (p < 0.001; p = 0.017). 24-h glycemic responses were similar irrespective of meal time. Participants with late chronotype consuming a high GI meal in the morning or evening showed similar 2-h postprandial (iAUC: 211 (± 110) vs. 207 (± 95) (mmol/L) × min, p = 0.9) and 24-h glycemic responses at both daytimes.

**Conclusions:**

Diurnal differences in response to a high GI meal are confined to those young adults with early chronotype, whilst those with a late chronotype seem vulnerable to both very early and late high GI meals. Registered at clinicaltrials.gov (NCT04298645; 22/01/2020).

**Supplementary Information:**

The online version contains supplementary material available at 10.1007/s00394-024-03372-4.

## Introduction

Accumulating evidence suggests that eating meals late in the evening affects postprandial (pp) glucose and insulin responses more adversely than consuming identical meals at early daytimes [1]. This is particularly pronounced for evening consumption of carbohydrate-rich meals with a high glycemic index (GI) among both healthy individuals [2, 3] and persons with impaired fasting glucose and/or impaired glucose tolerance [4]. Mechanistically, this phenomenon is likely attributable to the diurnal rhythm of glucose homeostasis characterized by a decrease in insulin secretion and/or sensitivity over the day resulting in lower glucose tolerance in the evening [5, 6]. Hence, the recent trend to shift main daily energy intake to later daytimes [7] is a public health concern and may contribute to the worldwide burden of type 2 diabetes [8].

Individuals with a late circadian phenotype, i.e. late chronotype, who habitually consume their main meals in the evening [9], may be particularly at risk for type 2 diabetes. A recent meta-analysis reported higher fasting blood glucose and HbA_1c_ levels as well as a higher risk for type 2 diabetes among healthy individuals with a late chronotype compared to individuals with an early chronotype [10]. Persons with a late chronotype are also more likely to experience discrepancies between their circadian rhythm and socially determined schedules such as early starting time of university/school [11]. Hence, for individuals with a late chronotype consumption of an early breakfast – due to social routines – could entail circadian misalignment (which characterizes a de-synchronized biological and behavioral cycle [5]).Meanwhile, persons with an early chronotype may be vulnerable to a late high GI meal due to both circadian misalignment and the above described circadian rhythmicity of glucose tolerance. Since the concurrence of elevated melatonin concentrations and food consumption may adversely affect glucose tolerance [12] it is of interest to investigate melatonin concentration in persons with different chronotypes (e.g. in routinely measured fasting samples).

To date, the diurnal glycemic response has not been investigated by chronotype yet. Therefore, this study addresses the hypothesis that a diurnal rhythm – with higher 2-h pp and 24-h glycemic response to a meal rich in carbohydrates from higher GI sources when consumed early in the morning (7 a.m.) or late in the evening (8 p.m.) may be discernible in persons with early chronotype, in whom late consumption may represent circadian misalignment. By contrast, we hypothesize that early consumption may entail circadian misalignment for persons with a late chronotype. Hence, we compared 2-h pp and 24-h glycemic responses in a cross-over trial conducted in two samples of students with either early or late chronotype.

## Research design and methods

### Study population

**Fig. 1:**
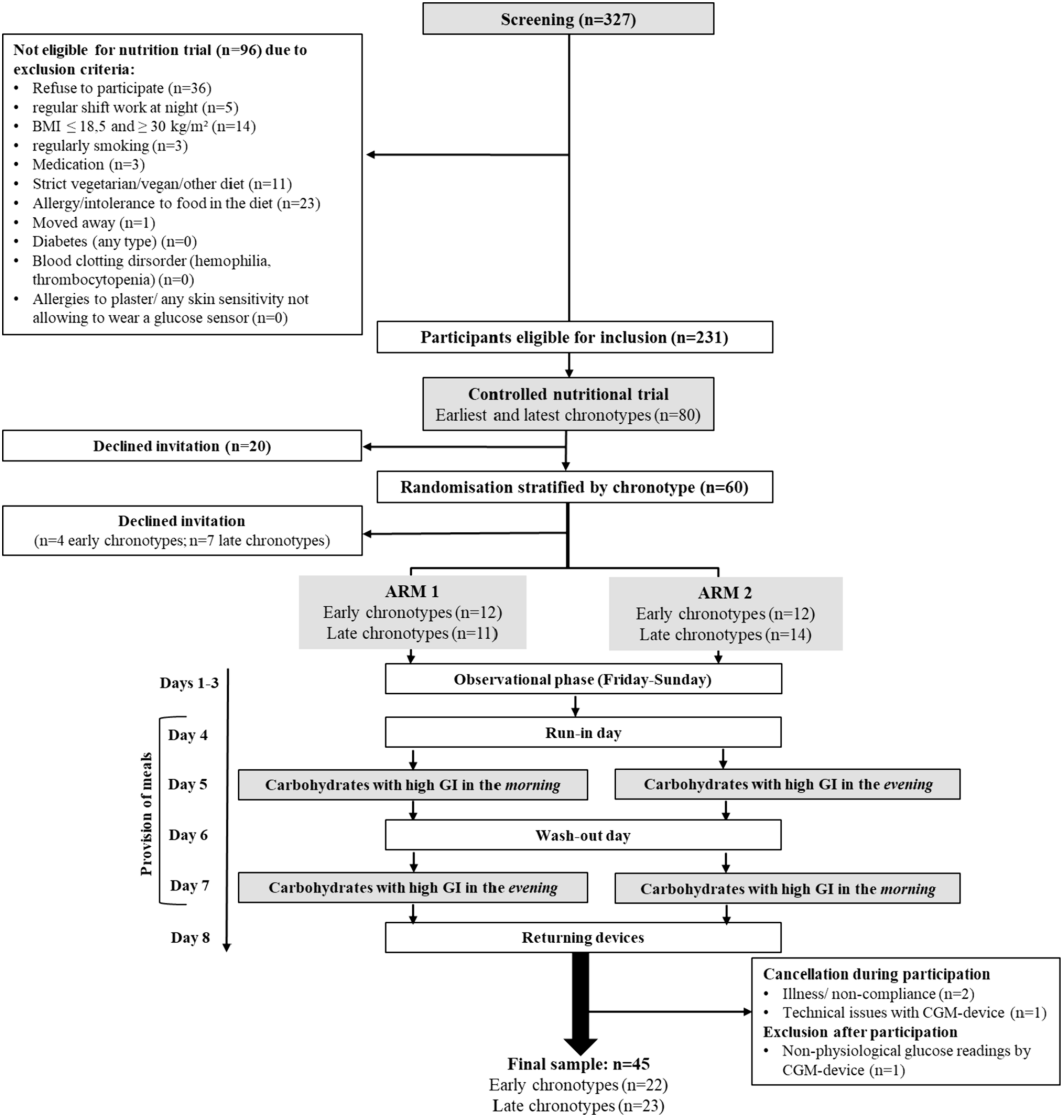
Flow chart of study participants and the procedure of the controlled nutrition trial. Of n = 327 screened students, n = 80 were invited to participate of which n = 46 completed the intervention while one participant was excluded due to non-physiological glucose recordings

For the Chronotype and Nutrition (ChroNu) study a screening of 327 students was conducted during September 2019 to January 2020, as described previously [11]. In brief, students aged 18–25 years at Paderborn University answered questionnaires on their chronotype and the timing of daily routines; body composition was measured. Exclusion criteria are listed in Fig. 1. Among the 231 students eligible for inclusion in the controlled nutritional trial (Fig. 1), those with the earliest (n = 40) and the latest (n = 40) chronotype were invited. Of these, 20 persons declined the invitation before and 11 individuals after randomization, i.e. they did not participate in the trial. During the trial, 3 further persons were excluded due to illness/non-compliance and technical issues with the continuous glucose monitoring device. Hence, 46 students completed the nutrition trial. Data from one participant were excluded for the analysis due to non-physiological glucose readings, resulting in a final sample of n = 45 for analysis. The trial was conducted at Paderborn University during September 2020 to December 2020. Informed consent was obtained from all participants prior to the trial. The study protocol was approved by the Ethics Committee of Paderborn University (16.05.2019) and was performed in accordance with the ethical standards laid down in the 1964 Declaration of Helsinki and its later amendments [13]. The trial was registered at clinicaltrials.gov (NCT04298645).

### Chronotype assessment

Chronotype was assessed both at screening and prior to the nutrition trial using the Munich ChronoType Questionnaire, which enquires about sleep time separately for work- and work-free days [14]. The individual chronotype was the midpoint of sleep between sleep-onset and sleep-offset and corrected for accumulated sleep debt (temporal difference in sleep duration between work and work-free day) during workdays (MSF_sc_).

### Design of the nutrition trial

On day 1, participants were asked to fill in online questionnaires via REDCap [15]. On day 4, i.e. after a 3-day observational phase, participants were randomized to the order of high GI (GI ≥ 70) meal consumption in the morning/evening (arm 1) or evening/morning (arm 2) on the intervention days (days 5 and 7), preceded by a run-in/wash-out day (days 4 and 6), respectively (Fig. 1). Participants were instructed to avoid consumption of legumes on day 3 to prevent any potential influences on the fasting glycemia values obtained in the morning of the run-in day [16]. In the morning of the run-in day, fasting blood sample was taken, and participants consumed breakfast and received their morning snack. Participants returned for a freshly prepared warm lunch and received consecutive study afternoon snack, dinner, breakfast, morning snack in labelled boxes for consumption at home until lunch on the next day, which was again provided at the study center. This schedule was maintained until day 7, when only afternoon snack and dinner were handed out after lunch. Participants were instructed to consume meals/snacks without a break at predefined times (Supplemental Tables 1, 2). On run-in/wash-out days, participants were instructed to consume their dinner before 9 p.m. to ensure a 10 h fasting period before the intervention day. Participants were asked to record the timing of meal/snack consumption, their activities, and sleep timing in a diary to corroborate compliance.

### Intervention

An identical high GI meal was provided in the morning (7 a.m.) or evening (8 p.m.), i.e. at times commonly imposed by social schedules, yet potentially causing circadian misalignment for late/early chronotypes [14]. The meal consisted of a Mars® bar, Cornflakes (Kellog´s^®^), low-fat milk (1,5%), and a soft pretzel (Ditsch®) resulting in an estimated meal GI of 72 (Supplemental Table 1). On the intervention days, this high-GI meal provided 35% of the daily amount of available carbohydrates (grams). All other meals/snacks on the intervention and run-in/wash-out days were designed to have an estimated medium GI between 46 and 59 (Supplemental Tables 1, 2), to avoid second meal effects [16]. On the run-in/wash-out days, lunch provided the largest proportion of available carbohydrates. Meal GI estimation was conducted according to a previously published procedure [17]. Food items with a published GI [18] were given preference to allow for a valid estimation of the meal GI particularly of the intervention meal. Hence, pretzels were used, i.e. the only tested German bread with a value GI > 70 [19]. If more than one published GI value was available, the mean of these values was assigned. The dietary GI of each meal/snack was calculated as the sum of glycemic load (GL) values of each food divided by the sum of their available carbohydrates (g)*100 [17].

Participants followed an isocaloric diet to maintain body weight. To this end, total energy expenditure was estimated individually based on resting energy expenditure using the formula by Harris & Benedict [20] multiplied by a physical activity level of 1.4 since participants were instructed to avoid (vigorous) physical activity. Participants were grouped into categories based on the total energy expenditure distribution of the study population: 1900 kcal, 2100 kcal, 2300 kcal, 2500 kcal, 2700 kcal, 2900 kcal. During the trial, participants were allowed to switch the TEE category once. The energy content of the provided meals was calculated using the nutrition programme DGExpert designed by the German Nutrition Society, which is based on the German food table (Bundeslebensmittelschlüssel) [21]. The diets of the intervention and the run-in/wash-out days were designed to comply with the recommendations of the German Nutrition Society to consume a diet rich in carbohydrates [22] and contained 14En% from protein, 30En% from fat, 53En% from available carbohydrates, and 3En% from dietary fiber. Noteworthy, the macronutrient distribution (En%) was similar on all study days. During the trial, participants were asked to consume the provided foods only and to abstain from consuming alcohol/alcohol-free drinks, caffeinated/decaffeinated beverages, and carbohydrate containing beverages. Participants were provided with a selection of teas containing < 0.3g carbohydrates/serving (200 mL).

### Outcomes

The primary outcome, on which the power calculation was based, was the 2-h difference in the incremental area under the curve (iAUC) while the further outcomes were the difference of iAUC and mean amplitude of glucose excursions (MAGE) over a time span of 24 h following the consumption of the high GI meal between morning and evening. Additionally, parameters describing glycemic variability (mean, standard deviation, highest, and lowest glucose value) were analyzed. During the study, glycemic responses were recorded using continuous glucose monitoring (G6, Dexcom, San Diego, CA, USA), which measures subcutaneous interstitial glucose concentrations resulting in mean glucose value every 5 min. The device was blinded during the trial (days 4–8).

### Corroboration of chronotype

During the trial, participants were asked to wear an accelerometer (E4 wristband, Empatica SRI, Italy) day and night to objectively monitor their activity and resting phases. Sleep and awake times during the trial were estimated based on movement recordings of the accelerometer and bedtimes entered in the diary, which the participants used to record their daily routines/activities during the study. Time of sleep onset and wake-up during the nutrition trial was averaged for days 4 to 7.

### Anthropometric and laboratory measurements

To monitor changes in anthropometry, body composition, i.e. visceral fat mass and skeletal muscle mass, was measured by using Bioimpedance Analysis (mBCA 515, SECA, Hamburg, Germany) on day 1 (in the afternoon) and day 8 (in the morning) (Supplementary Table 3). Waist circumference (cm) was measured midway between the lowest rib and the iliac crest. Body size was measured using an ultrasonic measuring station (seca 287 dp, Hamburg, Germany) from SECA. BMI was calculated by weight (kg)/height (m)^2^.

On day 4, venous blood samples were collected at 7 a.m. after ≥ 10 h overnight fast for measurement of glucose, insulin, lipids, and high-sensitivity C-reactive protein (hsCRP). Blood samples were centrifuged after 10 and 30 min. EDTA-plasma and serum samples were stored at -20 °C and shipped to the German Diabetes Center in Düsseldorf for analyses. Fasting plasma insulin was measured with a chemiluminescence immunoassay (Immulite 2000 xPi; Siemens, Erlangen, Germany). A clinical chemistry autoanalyzer (Cobas c-311; Roche, Mannheim, Germany) was used to measure fasting blood glucose (hexokinase reference method), triglycerides (TGs), i.e. lipoproteins, low-density cholesterol (LDLc), high-density cholesterol (HDLc), as well as plasma nonesterified fatty acids using enzymatic colorimetric assay, and hsCRP with the use of an immunoturbidimetric assay [23, 24]. Melatonin was subsequently measured by ELISA (sunrise, TECAN IBL International, Hamburg, Germany) for the 44 participants with sufficient serum material at Medizinische Laboratorien, Düsseldorf. HOMA-IR was calculated as (fasting blood insulin in µU/mL*fasting blood glucose in mmol/L)/22.5 [25].

### Characteristics on eating pattern

Habitual consumption (yes/no) and timing of meals/snacks were inquired separately for work- and work-free days. Non-consumption of breakfast/lunch/dinner was defined as skipping the corresponding meal.

### Sample size

Sample size estimation of expected difference in the 2-h pp difference in morning vs. evening iAUC (primary outcome) following the consumption of the high-GI intervention meal was based on data from Morris et al. [5]. They observed that the 2-h pp iAUC to a carbohydrate-rich meal was 913 ± 26 (SEM) (mmol/L) × min in the morning and 1,096 ± 17 (mmol/L) × min in the evening (values conservatively estimated from Fig. 4 [5]), i.e. differed by approx. 180 (mmol/L) × min. Hence, including a total of n = 8 participants would accordingly allow to detect a difference of < 180 (mmol/L) × min between the morning and evening meal with a power of 80% (PROC POWER, SAS University Edition) – using a standard deviation of 98 (mmol/L) × min (i.e. estimated from the more conservative SEM reported for morning consumption [5]). Based on previous experiences [26] we assumed a 15% drop-out rate, hence the estimated sample size per arm was n = 10 (n = 20 in total). Since we planned to perform this study in two separate samples with early and late chronotypes we aimed to include 40 persons in total. With an expected participation rate of 66%, our aim was to recruit 60 eligible participants. We estimated that a total of 300 students needed to be screened to identify the participants with the latest and earliest chronotype (10% each) identified as 20% of the participants with each the earliest and latest MSF_sc_ among the cohort.

### Randomization and masking

Due to the COVID19 pandemic fewer students were willing to participate. Hence n = 80 persons had to be invited in total. Of these, 60 participants initially accepted the invitation and were randomly assigned to arm 1 or arm 2 stratified by sex and chronotype with a block size = 4 considering 20 participants per strata [27] by JD (University of Bergen), Fig. 1. While the participants and researchers were not blinded to the study arm due to the nature of the study involving provision of meals, researchers were blinded to the participants´ chronotype.

### Calculations

For analysis of 2-h pp and 24-h iAUC trapezoidal rule ignoring areas below baseline was applied [28]. Baseline was calculated as the mean of glucose readings 5 min. before and (i) at time point of meal consumption (2-h-pp iAUC) and (ii) at 7 a.m. (24-h-pp iAUC) in accordance with GI testing guidelines [28]. MAGE was calculated by use of the validated EasyGV program [29]. 24-h glycemic response and variability covers a timespan from the intervention day (7 a.m.) until 7 a.m. of the following day.

### Statistical analyses

Descriptive data are reported as mean ± SD if normally distributed, otherwise as median (Q1, Q3). Categorical variables are shown as percentages. As this study aimed to compare effects on 2-h pp and 24-h glycemic response following high-GI meal consumption in the morning vs. evening within both a group of early and a group of late chronotypes, multilevel linear regression was applied including chronotype and time of consumption (morning or evening) as fixed effects and participant as a random effect. By nature, these models consider the dependence between repeated measures within a person (PROC MIXED in SAS). Beta-coefficients (and 95% confidence limits) for the time variable are presented as estimates of the mean differences between morning and evening consumption. To facilitate interpretation differences are presented as evening minus morning consumption. The variable 24-h standard deviation was log-transformed to achieve normal distribution of the model residuals. The beta-coefficient for this variable was retransformed and differences represent percent differences between evening and morning consumption [30].

Only few participants exhibited > 1 standard deviation during 2-h pp interval (n = 6 early; n = 8 late chronotypes) allowing for a calculation of 2-h MAGE, hence, only 24-h MAGE was analysed.

To examine whether melatonin concentrations (available from routinely measured fasting levels only) may be related to glucose tolerance in this study correlation and linear regression were performed relating melatonin concentrations to the primary outcome 2-h pp glucose iAUC following the high-GI intervention meal. Since melatonin measurements were only available from fasting (i.e. morning) blood samples this analysis was confined to 2-h pp glucose iAUC after morning high-GI meal consumption. Statistical analyses were performed using SAS procedures (SAS version 9.4; SAS Institute, Cary, NC, USA) considering *p*-values < 0.05 as statistically significant except for analyses of interactions where p-values < 0.1 were considered significant [31].

## Results

All results are presented stratified by chronotype in accordance with the study design. This was underpinned by interactions of chronotype with the effects of the intervention (morning vs. evening) on 2-h pp (mean (p = 0.09) and highest glucose values (p = 0.06)) and 24-h (highest glucose values (p = 0.04), standard deviation (p = 0.02), and MAGE (p = 0.098)) glucose response variables (all p < 0.1, which is regarded significant for interactions [31]).

### Characteristics of the study population

Participants were on average 22 years old and healthy as indicated by their body composition and physiological data (Table 1). Persons with early and late chronotypes differed in their mean morning melatonin levels (27.4 vs. 36.0 ng/L) measured from blood samples withdrawn at 7 a.m. MSFsc differed by approximately 1:54 h:min between early and late chronotypes. Similarly, time when falling asleep and waking up were notably different between the two chronotype groups. Both chronotypes had to wake up earlier than normal during the nutrition trial.

**Table 1:** Characteristics of the two study populations (early and late chronotypes)

	Early chronotypes (n = 22)	Late chronotypes (n = 23)
Anthropometric and laboratory characteristics		
Female sex, n (%)	14 (64)	12 (52)
Age, years	22 (21; 23)	22 (21; 24)
BMI, kg/m^2^	22.4 (± 2.2)	22.5 (± 2.6)
Waist circumference, m	0.7 (0.7; 0.8)	0.8 (0.8; 0.9)
Visceral fat mass, L	0.4 (0.3; 0.6)	0.7 (0.4; 1.3)
Skeletal muscle mass, kg	23.2 (20.1; 28.4)	24.9 (21.2; 31.3)
Fasting blood glucose, mmol/L	5.1 (± 0.4)	5.2 (± 0.3)
Fasting insulin, µU/mL	6.9 (± 2.7)	7.3 (± 3.9)
HOMA-IR	1.6 (± 0.6)	1.7 (± 0.9)
Melatonin, ng/L	27.4 (22.7; 38.1)	36.0 (29.4; 57.1)
hsCRP, mg/dL	0.1 (0.0; 0.1)	0.0 (0.0; 0.1)
Non-esterified fatty acids, µmoL/L	359 (231; 589)	409 (309; 524)
Triglycerides, mg/dL	98 (± 42)	99 (± 45)
HDLc, mg/dL	61 (± 13)	64 (± 15)
LDLc, mg/dL	104 (± 25)	97 (± 25)
Circadian characteristics and habitual meal/snack consumption		
Chronotype MSFsc (o’clock)		
At screening	3:26 (2:55; 3:38)	6:00 (5:35; 6:23)
At intervention^1^	3:54 (3:15; 4:18)	5:50 (5:10; 6:25)
Time when falling asleep (o’clock)		
Workdays^1^	22:50 (22:25; 23:11)	1:00 (00:15; 1:10)
Work-ree days^1^	23:32 (23:10; 00:00)	2:00 (1:20; 2:10)
During nutrition trial^2^	22:46 (22:21; 23:12)	23:48 (23:31; 00:47)
Wake-up time (o’clock)		
Workdays^1^	7:00 (6:30; 7:30)	9:00 (8:00; 9:05)
Work-free days^1^	8:00 (7:15; 8:30)	10:00 (9:00; 10:45)
During nutrition trial^2^	6:36 (6:16; 6:44)	7:07 (6:41; 7:26)
Breakfast timing (o’clock)^1^		
Workdays	7:52 (7:00; 8:15)	10:00 (9:00; 10:00)
Work-free days	9:00 (8:37; 9:30)	11:00 (10:00; 12:00)
Lunch timing (o’clock)^1^		
Workdays	13:00 (12:30; 13:30)	14:00 (13:00; 14:00)
Work-free days	13:30 (12:30; 14:00)	14:00 (13:30; 15:00)
Dinner timing (o’clock)^1^		
Workdays	19:00 (18:30; 19:00)	19:30 (19:00; 20:00)
Work-free days	19:00 (18:00; 19:00)	19:30 (18:30; 20:46)
Breakfast skipping (n (%))^1^		
Workdays	4 (18)	5 (22)
Work-free days	2 (9)	4 (17)
Lunch skipping (n (%))^1^		
Workdays	1 (5)	4 (17)
Work-free days	7 (37)	7 (30)
Snacking in the morning (n (%))^1^		
Workdays	8 (36)	5 (22)
Work-free days	5 (23)	4 (17)
Snacking in the evening (n (%))^1^		
Workdays	2 (9)	6 (26)
Work-free days	6 (27)	10 (43)

*hsCRP* high-sensitivity C-reactive protein, *HDLc* high-density cholesterol, *LDLc* low-density cholesterol, *MSFsc* midpoint of sleep corrected

^1^Estimated time of the past 4 weeks before intervention [41]. Note that lectures were still held online at university. ^2^Days 4 to 7. Dinner skipping was minimal (i.e. < 5%). Data are frequencies, means ± standard deviation, or medians (Q1, Q3)

### Glycemic response of participants with early chronotype

**Fig. 2:**
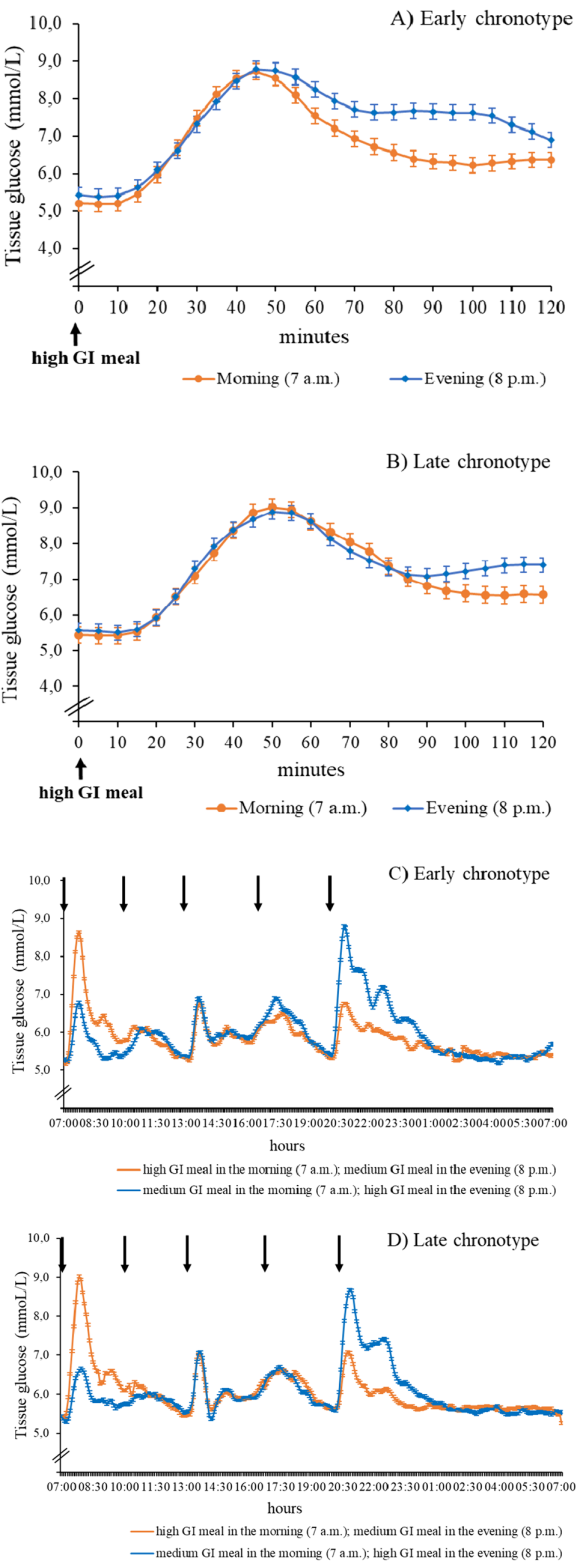
Mean glucose levels (± standard error) 2-h pp following a high GI meal in the morning (blue) and in the evening (orange) (panel (**A**,**B**)) and 24-h distribution (7–7 a.m.; panel (**C**,**D**)) among participants with early and late chronotype (n = 22; n = 23, respectively). 2-h pp mean glucose values were significantly higher after high GI meal in the evening than in the morning among early chronotypes only (p < 0.001). Black arrows indicate meal/snack consumption

For persons with an early chronotype, the *2-h pp glycemic response* was lower when the intervention high-GI meal was consumed early in the morning compared to late in the evening (195 (± 91) vs. 234 (± 92) (mmol/L) x min, p = 0.042) **(**Table 2). Similarly, the mean (p ≤ 0.001) and the lowest 2-h pp glucose values (p = 0.017) were lower in the morning. Figure 2A illustrates that glucose levels increased similarly within the first 50 min. pp, but remained elevated for a longer period when the intervention meal was consumed in the evening. Additionally, high GI meal consumption in the evening resulted in a higher standard deviation of the *24-h responses* (p = 0.001). Figure 2C shows that evening glycemic responses to the high GI meal remained elevated for longer (until 1 a.m.) than evening responses to the medium GI meal.

**Table 2:** Glycemic response parameters to a high GI meal consumed in the morning and in the evening by chronotype group

Glycemic response parameters	Early chronotype (n = 22)				Late chronotype (n = 23)			
2 h pp response after high GI meal consumed	Morning (7 a.m.)	Evening (8 p.m.)	Difference (95% CI) evening versus morning^1^	p	Morning (7 a.m.)	Evening (8 p.m.)	Difference (95% CI) evening versus morning^1^	p
iAUC((mmol/L) x min)	195 (± 91)	234 (± 92)	40 (2; 77)	**0.042**	211 (± 110)	207 (± 95)	− 4 (− 55; 48)	0.888
Mean glucose value (mmol/L)	6.75 (± 0.91)	7.32 (± 0.83)	0.57 (0.29; 0.86)	** < 0.001**	7.12 (± 1.04)	7.28 (± 0.68)	0.16 (− 0.20; 0.53)	0.362
Highest glucose value (mmol/L)	9.04 (± 1.42)	9.57 (± 1.37)	0.52 (0.07; 1.11)	0.080	9.67 (± 1.58)	9.39 (± 1.17)	− 0.28 (− 0.92; 0.35)	0.365
Lowest glucose value (mmol/L)	4.80 (± 0.71)	5.19 (± 0.54)	0.38 (0.08; 0.69)	**0.017**	5.00 (± 0.61)	5.33 (± 0.53)	0.32 (0.05; 0.59)	**0.024**
Standard deviation (mmol/L)	1.34 (± 0.44)	1.42 (± 0.50)	0.07 (-0.18; 0.33)	0.551	1.52 (± 0.51)	1.30 (± 0.41)	− 0.21 (− 0.47; 0.04)	0.098

24-h glycemic response on days with high GI meal consumed	Morning (7 a.m.–7 a.m.)	Evening (7 a.m.–7 a.m.)	Difference (95% CI) evening versus morning^1^		Morning (7 a.m.–7 a.m.)	Evening (7 a.m.–7 a.m.)	Difference (95% CI) evening versus morning^1^	
iAUC ((mmol/L) x min)	1004 (± 399)	1071 (± 289)	67 (− 114; 248)	0.452	962 (± 368)	987 (± 352)	26 (− 13; 190)	0.748
Mean glucose value (mmol/L)	5.87(± 0.59)	5.97 (± 0.58)	0.11 (0.00; 0.21)	0.052	6.06 (± 0.38)	6.05 (± 0.43)	− 0.01 (− 0.12; 0.11)	0.876
Highest glucose value (mmol/L)	9.19 (± 1.20)	9.68 (± 1.26)	0.48 (− 0.04; 1.00)	0.069	9.74 (± 1.46)	9.42 (± 1.17)	− 0.32 (− 0.89; 0.24)	0.251
Lowest glucose value (mmol/L)	4.15 (± 0.67)	4.44 (± 0.65)	0.29 (− 0.06; 0.63)	0.098	4.46 (± 0.65)	4.51 (± 0.46)	0.05 (− 0.24; 0.34)	0.719
Standard deviation (mmol/L)	0.79 (± 0.15)	0.92 (± 0.21)	16% (7%; 24%)^2^	**0.001**	0.87 (± 0.24)	0.86 (± 0.20)	0.2% (− 10%; 11%)^2^	0.972
MAGE (mmol/L)	2.25 (1.74; 2.46)	2.17 (1.88; 2.65)	0.17 (− 0.12; 0.45)	0.233	2.31 (1.87; 2.77)	2.23 (1.77; 2.59)	− 0.07 (− 0.42; 0.27)	0.661

Data are means ± standard deviation or medians (Q1, Q3) calculated from the individual iAUC, mean, highest and lowest value as well as the intra-individual standard deviation obtained during 2-h pp or 24-h pp each individual. MAGE was analyzed for 24 h-period only due to low number of participants with > 1 standard deviation during 2-h pp interval

*n*, sample size, *iAUC* incremental area under the curve, *MAGE* mean amplitude of glucose excursions, *CI* confidence interval

^1^Difference estimated from multilevel linear regression (ß coefficients) ^2^Percentage difference evening vs morning as estimated from log-transformed variable. Significant P-values (< 0.05) are marked in bold

### Glycemic response of participants with late chronotype

Participants with a late chronotype showed comparable *2-h pp glycemic responses* and variability after consumption of the high GI meal early in the morning and late in the evening; only the lowest 2-h pp glucose value was higher in the evening (5.00 (± 0.61) vs. 5.33 (± 0.53) (mmol/L) × min; p = 0.024) **(**Table 2**,** Fig. 2B). Similarly, with respect to *24-h glycemic responses* no significant differences were seen (Table 2, (Fig. 2D). As with early chronotypes, evening glucose levels remained elevated for ~ 5 h after the high GI evening meal.

### Analysis of melatonin

Analysis for the sample revealed that the morning melatonin level was associated with the 2-h pp glycemic response to the high GI meal consumed in the morning (r = 0.33; p = 0.03) (Supplementary Fig. 1).

### Anthropometric analysis

Among both groups, BMI and waist circumference were somewhat lower when measured after the intervention in the morning of day 8 in comparison to measurements on day 1 in the afternoon (Supplementary Table 3).

## Discussion

This is the first study examining the 2-h and 24-h glycemic responses to high GI meals consumed early in the morning (7 a.m.) and late in the evening (8 p.m.) in two selected samples of young adults with early and late chronotype, confirmed with two different methods—the MCTQ (questionnaire) and accelerometers. Importantly, our study suggests that diurnal differences in 2-h pp glycemia – whilst seen among students with early chronotype – may not hold true for young adults with a late chronotype.

### Findings among students with late chronotype

Of note, individuals with late chronotype showed no difference in 2-h and 24-h glycemic response to morning and evening high GI meals. It could be argued that our observation for students with late chronotype may be due to an emerging insulin resistance, which may subsequently have contributed to some loss in circadian rhythmicity. In fact, among persons with prediabetes, higher HOMA-IR levels were associated with a reduced circadian rhythmicity [32], yet the authors speculate that this was largely a consequence of a loss in circadian rhythmicity caused by higher BMI levels. Whilst in our study, pp insulin concentrations were not investigated, fasting HOMA-IR was similar among adults with late or early chronotype and both groups were on average of normal weight (Table 1). Hence, this argues against the idea that metabolic abnormalities may have contributed to a loss in circadian rhythmicity.

By contrast, the timing of the high GI meal at 7 a.m. in this controlled trial was designed to interfere with circadian rhythmicity among persons with a late chronotype (MSF_sc_ = 5:50 a.m.), who habitually consume breakfast at 11 a.m. on work-free days (Table 1). Meal consumption against the inner clock results in a conflict between the rhythm of peripheral clocks and the central pacemaker of the diurnal rhythm located in the suprachiasmatic nucleus [33]. Consequently, meal consumption induces peripheral signals that activate organs and tissues, while the central pacemaker still signals the biological night [33]. Of note, higher morning melatonin levels among persons with late chronotype underpins our assumption of circadian misalignment, since melatonin concentrations are regulated by the suprachiasmatic nucleus and follow a circadian rhythm (i.e. increasing ~ 2 h before biological sleep, peaking in the first half of sleep phase, and declining continuously over ~ 2–3 h after habitual wake-up [12, 34, 35]). Higher melatonin levels have been shown to inhibit glucose-stimulated insulin secretion through binding to melatonin-receptors in the pancreatic beta cells and/or decrease insulin sensitivity, i.e. affecting glucose tolerance [12]. Hence, this may have contributed to the absence of lower glycemic response in the morning among persons with a late chronotype. Of note, our subsequent analysis confirmed that higher melatonin levels drawn at ~ 7 a.m. at day 1 of the intervention were associated with higher pp glycemic responses at 7 a.m. on the intervention days 5 or 7. In line with our results, another study among healthy individuals reported higher morning melatonin levels in response to sleep-restriction by ~ 2.5 h compared to a habitual sleep phase (wake-up time 5:30 a.m. vs. 8 a.m.) [34]. In that study, sleep restriction resulted in increased pp glucose response following breakfast at 6:15 a.m. when compared to 8:45 a.m. after habitual sleep duration [34]. Taken together, our data indicate that a high GI meal consumed early in the morning may be similarly detrimental to its consumption in the evening among persons with a late chronotype, hence supporting our hypothesis of a circadian misalignment.

### Findings among students with early chronotype

In line with the diurnal decline in glucose homeostasis [1, 5], the hypothesized differences in 2-h glycemic response to the high GI meal consumed in the morning and evening were observed among adults with early chronotype. Noteworthy, differences emerged after 50 min (Fig. 2A), suggesting that a higher early-phase insulin response in the morning may have led to a faster decrease of glucose concentrations than in the evening when beta cell responsiveness is reduced [6]. Of note, higher mean and 24-h glucose values emphasize the lasting effect of a high GI meal late in the evening on glucose homeostasis. Similarly, a study reported sustained adverse influences of a late evening meal consumption (9 p.m.) compared to early evening meal time (6 p.m.) among healthy adults on mean diurnal glucose responses [36]. In our study, evening meal timing (8 p.m.) was later than self-reported habitual dinner time (median 7 p.m.) among early chronotypes and only ~ 3 h apart from their habitual time of falling asleep. Hence, timing of the evening meal may additionally represent some degree of circadian misalignment, which has been shown to lower glucose tolerance mainly by reduced insulin sensitivity [37] independently of diurnal rhythms [5]. Another study also reported higher glucose levels after late evening meal consumption (10 p.m.) compared to the early evening meal time (6 p.m.) among young healthy adults accustomed to a bedtime between 10 p.m. and 1 a.m. [38]. The authors attributed this difference to circadian misalignment acting primarily among participants who habitually sleep at early daytime [38].

We speculate that previous studies – often entailing study visits early in the morning – were predominantly performed among persons with early chronotype. Further studies are needed to examine whether a diurnal difference between morning and evening consumption may also be discernible among adults with late chronotypes when comparing glycemic response to a high GI meal consumed late in the morning (e.g. 11 a.m.) compared to its consumption in the evening.

Of note, for both adults with early and late chronotype glucose levels remained elevated for ~ 5 h after the high GI meal consumed at 8 p.m. (Fig. 2C,D). Previous studies reported higher glucose levels after earlier (7 p.m.) vs. later (10:30 p.m.) dinner consumption that were maintained up to 5 h until night and thus interfered with the participants´ sleep phase [38, 39].

The public health implications of our study results are thus twofold: First, carbohydrate-rich meals with a high GI should best be avoided particularly at later daytimes [2, 5] regardless of chronotype. Second, socially determined schedules in institutions such as school/university need to enable more flexibility in the timing of breakfast particularly for persons with late chronotype. This group presently either skips breakfast (with potential adverse consequences [40]) or consumes breakfast very early (as in our study), hence increasing the risk of circadian misalignment. Since an early breakfast may only be beneficial for adults with early chronotype, social schedules at institutions should allow breaks for a breakfast later in the morning.

### Strengths and limitations

The main strength of this study is the study population with selected early and late chronotypes based on an initial screening using a validated questionnaire [41]. This resulted in a notable difference in MSF_sc_ by almost 2 h. In addition, both wake-up time estimated from the data recorded by the accelerometer and mean melatonin levels confirm our distinction between the early and late chronotype group.

There are some limitations to this study. First, this study was a priori designed to compare effects on glycemic response following high GI meal consumption in the morning vs. evening within two chronotype strata. This was based on the rather explorative purpose of this study to examine whether either early or late chronotypes are vulnerable to eating meals against their inner clock and secondly on pragmatic considerations since conduction of a study powered for analysis of an interaction would have quadrupled the sample sizes in each group, and hence required a screening of an unfeasibly large sample (approximately 1200 students) [42]. Indeed, morning or evening iAUC did not differ significantly between early and late chronotypes (data not shown), yet this was not the aim of this study and may largely reflect the lack of power for this comparison. Nonetheless interaction tests confirm differences between early and late chronotypes for the difference between morning and evening consumption for selected outcomes (see “Methods”). Despite the fact that we aimed to include persons with extreme early and late MSF_sc_ [41], the study population comprises individuals with merely moderately early or moderately late chronotype. Hence, this potentially limited our possibility to detect more extreme glycemic responses. Another limitation concerns the absence of measured glucose homoeostasis associated hormones such as insulin and those that display circadian rhythmicity, e.g. cortisol and ghrelin, or those involved in the synchronization of the central circadian rhythm with the peripheral tissues such as leptin, adipokines, and incretins [33]. Also, data on the individual circadian rhythm at the time of the intervention are lacking. Moreover, we were not able to perform an intention-to-treat analysis because the persons who declined participation did not participate in any of the study visits after randomization. However, since dropout occurred in all groups, selection bias towards a null effect is improbable, as it would only be possible if dropout individuals were expected to show an opposite effect. Finally, this study was conducted among young and healthy university students, thus our results may not be generalizable to older or less healthy people.

## Conclusions

Glycemic responses to a high GI meal were higher in the evening than in the morning among adults with early chronotype, in agreement with the concurrence of circadian deterioration of glucose homeostasis and late evening meal consumption. Conversely, individuals with late chronotype showed no diurnal differences suggesting vulnerability to high GI meal consumption both in the very early morning and late in the evening. Further studies are needed to replicate our findings and to examine the physiological relevance of the observed differences.

### Supplementary Information

Below is the link to the electronic supplementary material.

Supplementary file1 (DOCX 78 KB)

## Data Availability

Data described in the manuscript, code book, and analytic code will be made available upon request pending [formal data sharing agreement].
