# Supplementary material for: Glycemic response to meals with a high glycemic index differs between morning and evening: a randomized cross-over controlled trial among students with early or late chronotype
Source: Eur J Nutr. 2024 Apr 12;63(5):1593–604. doi: 10.1007/s00394-024-03372-4 (PMC11329680; doi:10.1007/s00394-024-03372-4)
Supplement: Supplementary file 1 — Supplementary file1 (DOCX 78 KB) [file 394_2024_3372_MOESM1_ESM.docx]

**Glycemic response to meals with a high glycemic index differs between morning and evening - a randomized cross-over controlled trial among students with early or late chronotype**

Bianca Stutz^1^, Bettina Krueger^1^, Janina Goletzke^1^, Nicole Jankovic^2^, Ute Alexy^2^, Christian Herder^3 4 5^, Jutta Dierkes^6^, Gabriele Berg-Beckhoff^7^, Rasmus Jakobsmeyer^8^, Claus Reinsberger^8^, Anette E. Buyken^1*^

^1^ Faculty of Sciences, Institute of Nutrition, Consumption and Health, Paderborn University, Paderborn, Germany

^2^ Nutritional Epidemiology, Department of Nutrition and Food Sciences, Rheinische Friedrich-Wilhelms-University Bonn, DONALD study centre, Dortmund, Germany

^3^ Institute for Clinical Diabetology, German Diabetes Center (DDZ), Leibniz Center for Diabetes Research at Heinrich Heine University Düsseldorf, Düsseldorf, Germany

^4^ German Center for Diabetes Research (DZD), Partner Düsseldorf, München-Neuherberg, Germany

^5^ Department of Endocrinology and Diabetology, Medical Faculty and University Hospital Düsseldorf, Heinrich Heine University Düsseldorf, Düsseldorf, Germany

^6^ Department of Clinical Medicine Center, University of Bergen, Bergen, Norway

^7^ The Faculty of Health Sciences, Department of Public Health, University of Southern Denmark, Esbjerg, Denmark

^8^ Faculty of Sciences, Institute of Sports Medicine, Paderborn University, Paderborn, Germany

***Corresponding author:**

Anette E. Buyken, Faculty of Natural Sciences, Institute of Nutrition, Consumption and Health, Paderborn University, Warburger Strasse 100, 33098 Paderborn, Germany

Telephone number: +495251 603756

E-mail: [anette.buyken@uni-paderborn.de](mailto:anette.buyken@uni-paderborn.de)

**SUPPLEMENTAL TABLE 1:** Meal plan for the high GI intervention days 5+7 – example for the 2100 kcal group

| **Meals and instructed times of consumption** | **GI** | **GL^1^** | **Amount [g]** | **Kcal** | **Protein [g]** | **Fat [g]** | **Av. carbohydrates [g]** | **Fiber [g]** | **References** |
| --- | --- | --- | --- | --- | --- | --- | --- | --- | --- |
| **Breakfast (7:00 a.m.)/dinner (8:00 p.m.)** |  |  |  |  |  |  |  |  |  |
| Cornflakes (Kellog´s) | 81 | 29 | 42 | 161 | 3 | 0 | 35 | 1 | Mean of five studies [18] |
| Milk, 1,5 % fat content | 30 | 2 | 170 | 81 | 6 | 3 | 8 | 0 | Mean of milk, reduced fat (three studies) and milk, skim/low-fat [18] |
| Soft pretzel (Ditsch) | 80 | 23 | 60 | 165 | 5 | 2 | 29 | 2 | [19] |
| Mars® bar | 65 | 16 | 35 | 158 | 1 | 6 | 25 | 0 | Mean of 2 studies [18] |
| ***Meal GI^2^/GL^3^*** | ***72*** | ***70*** |  |  |  |  |  |  |  |
| **Morning snack (9:30-10:30 a.m.)** |  |  |  |  |  |  |  |  |  |
| Walkers Highland Oatcakes | 57 | 12 | 36 | 168 | 4 | 7 | 21 | 2 | [18] |
| Apple slices | 39 | 5 | 95 | 61 | 0 | 0 | 14 | 2 | [18] |
| ***Meal GI^2^/GL^3^*** | ***50*** | ***17*** |  |  |  |  |  |  |  |
| **Lunch^4^ (1:00 p.m.)** |  |  |  |  |  |  |  |  |  |
| Spaghetti (Transgourmet) | 48 | 24 | 72 | 263 | 9 | 2 | 50 | 2 | [18] |
| Chicken breast | 0 | 0 | 90 | 93 | 21 | 1 | 0 | 0 |  |
| Red pepper / Capsicum | 52 | 1 | 45 | 11 | 0 | 0 | 1 | 1 | Mean of green peas, sweet corn, carrots, pumpkin [18] |
| Carrots, diced | 41 | 1 | 45 | 18 | 0 | 0 | 3 | 1 | mean of two studies [18] |
| Tomato sauce^5^ | 32 | 1 | 146 | 54 | 3 | 0 | 8 | 2 | [18] |
| Olive oil | 0 | 0 | 15 | 133 | 0 | 15 | 0 | 0 |  |
| ***Meal GI^2^/GL^3^*** | ***48*** | ***31*** |  |  |  |  |  |  |  |
| **Afternoon snack (4:00-5:00 p.m)** |  |  |  |  |  |  |  |  |  |
| Belvita biscuits, milk&cereals | 45 | 9 | 31 | 139 | 2 | 4 | 21 | 2 | [18] |
| Kiwi slices | 58 | 7 | 125 | 70 | 1 | 1 | 11 | 5 | [18] |
| ***Meal GI^2^/GL^3^*** | ***50*** | ***16*** |  |  |  |  |  |  |  |
| **Dinner (8:00 p.m.)/Breakfast (7:00 a.m.)** |  |  |  |  |  |  |  |  |  |
| Bread (Lieken Urkorn, Paderborner) | 62 | 21 | 80 | 174 | 5 | 1 | 34 | 5 | [19] |
| Gouda cheese, 50% fat content | 0 | 0 | 48 | 172 | 12 | 14 | 0 | 0 |  |
| Butter | 0 | 0 | 16 | 117 | 0 | 13 | 0 | 0 |  |
| Cucumber slices | 52 | 0 | 55 | 5 | 0 | 0 | 1 | 0 | Mean of green peas, sweet corn, carrots, pumpkin [18] |
| Carrot sticks | 37 | 1 | 45 | 18 | 0 | 0 | 3 | 1 | mean of two studies [18] |
| Apple slices | 39 | 4 | 70 | 41 | 0 | 0 | 9 | 1 | [18] |
| ***Meal GI^2^/GL^3^*** | ***56*** | ***16*** |  |  |  |  |  |  |  |
| **Estimated GI/GL on intervention day** | **58** | **160** |  |  |  |  |  |  |  |
| **Total^6^** |  |  |  | **2100** | **74** | **70** | **274** | **29^7^** |  |
| **Total % energy** |  |  |  |  | **14** | **30** | **53** | **3** |  |

Abbreviations: g, grams; kcal, kilocalorie; G, glycaemic index; GL, glycemic load. ^1^Glycemic load= (g available carbohydrates*GI)/100; ^2^dietary GI= (sum of GL/ g available carbohydrates)*100; ^3^dietary GL= sum of GL; ^4^ Lunch was freshly cooked. Nutritional and GI values refer to the uncooked condition; ^5^Tomato sauce was self-made: canned tomatoes (mashed) (GI=52, GL= 2; mean of green peas, sweet corn, carrots, pumpkin [18], tomato puree (GI=52, GL=1; mean of green peas, sweet corn, carrots, pumpkin [18], sucrose (GI=56, GL=1; mean of six studies (16), vegetable broth, Italian herb mix (dry), salt, and pepper (all GI=0, GL=0); ^6^ total sum deviates from individual data listed due to rounding of decimals. ^7^ 2 kcal account for 1g fiber to achieve 100% total energy

**SUPPLEMENTAL TABLE 2:** Meal plan for the run-in/wash-out day – example for the 2100 kcal group:

| **Meals and instructed times of consumption** | **GI** | **GL^1^** | **Amount [g]** | **Kcal** | **Protein [g]** | **Fat [g]** | **Av. carbohydrates [g]** | **Fiber [g]** | **References** |
| --- | --- | --- | --- | --- | --- | --- | --- | --- | --- |
| **Breakfast (until 12:30 a.m.)** |  |  |  |  |  |  |  |  |  |
| Alpen Original Müsli | 55 | 32 | 88 | 332 | 10 | 5 | 58 | 7 | [18] |
| Milk, 3,8% fat content | 31 | 2 | 165 | 109 | 5 | 6 | 8 | 0 | mean of seven studies milk, full-fat [18] |
| Orange | 37 | 4 | 115 | 65 | 1 | 0 | 12 | 3 | Mean of two studies [18] |
| ***Meal GI^2^/GL^3^*** | ***50*** | ***39*** |  |  |  |  |  |  |  |
| **Morning snack (until 12:30 a.m.)** |  |  |  |  |  |  |  |  |  |
| Walkers Highland Oatcakes | 57 | 8 | 24 | 112 | 3 | 5 | 14 | 1 | [18] |
| Grapes | 59 | 10 | 120 | 83 | 1 | 0 | 18 | 2 | [18] |
| ***Meal GI/GL*** | ***58*** | ***18*** |  |  |  |  |  |  |  |
| **Lunch^4^ (1:00 p.m.)** |  |  |  |  |  |  |  |  |  |
| Basmati rice, Oryza Himalaya | 62 | 37 | 80 | 285 | 7 | 1 | 60 | 3 | [18] |
| Zucchini | 52 | 1 | 80 | 18 | 2 | 0 | 2 | 1 | mean of green peas, sweet corn, carrots, pumpkin [18] |
| Sweet corn, canned | 52 | 2 | 40 | 35 | 1 | 1 | 5 | 1 | mean of 5 studies [18] |
| Tomato sauce^5^ | 52 | 1 | 158 | 48 | 3 | 0 | 6 | 2 | [18] |
| Feta cheese | 0 | 0 | 40 | 115 | 7 | 10 | 0 | 0 |  |
| Olive oil | 0 | 0 | 10 | 10 | 0 | 10 | 0 | 0 |  |
| **Dessert** |  |  |  |  |  |  |  |  |  |
| Plain yoghurt, 3,8 % fat content | 19 | 1 | 110 | 80 | 5 | 4 | 6 | 0 | mean of four studies, natural yoghurt [18] |
| Raspberries | 51 | 2 | 90 | 40 | 1 | 0 | 4 | 4 | mean of blueberries wild; grapes, black, strawberries [18] |
| Honey | 61 | 5 | 11 | 34 | 0 | 0 | 8 | 0 | mean of 17 studies [18] |
| ***Meal GI/GL*** | ***57*** | ***52*** |  |  |  |  |  |  |  |
| **Afternoon snack (until 9:00 p.m.)** |  |  |  |  |  |  |  |  |  |
| Belvita biscuits, milk & cereals | 45 | 8 | 25 | 112 | 2 | 4 | 17 | 2 | [18] |
| Banana | 59,6 | 10 | 130 | 81 | 1 | 0 | 17 | 2 | mean of three studies [18] |
| ***Meal GI/GL*** | ***52*** | ***18*** |  |  |  |  |  |  |  |
| **Dinner (until 9:00 p.m.)** |  |  |  |  |  |  |  |  |  |
| Couscous, cooked | 65 | 21 | 50 | 170 | 6 | 1 | 32 | 3 | mean of 3 studies with identical preparation method [18] |
| Red pepper / capsicum | 52 | 1 | 45 | 10 | 0 | 0 | 1 | 1 | mean of green peas, sweet corn, carrots, pumpkin [18] |
| Tomato | 52 | 1 | 45 | 9 | 0 | 0 | 1 | 1 | mean of green peas, sweet corn, carrots, pumpkin [18] |
| Eggs, boiled | 0 | 0 | 80 | 109 | 9 | 7 | 1 | 0 |  |
| Cucumber | 52 | 0 | 35 | 4 | 0 | 0 | 0 | 0 | mean of green peas, sweet corn, carrots, pumpkin [18] |
| Avocado | 0 | 0 | 70 | 97 | 1 | 9 | 2 | 3 |  |
| Dressing^6^ | 0 | 0 | 14 | 62 | 0 | 7 | 0 | 0 |  |
| ***Meal GI/GL*** | ***58*** | ***22*** |  |  |  |  |  |  |  |
| **Estimated GI/GL on run-in/wash-out day** | **55** | **150** |  |  |  |  |  |  |  |
| **Total^7^** |  |  |  | **2100** | **65** | **72** | **274** | **36^9^** |  |
| **Total % energy^8^** |  |  |  |  | **13** | **30** | **53** | **3** |  |

Abbreviations: g, grams; kcal, kilocalorie; GI, glycaemic index; GL, glycemic load. ^1^Glycemic load= (g available carbohydrates*GI)/100; ^2^dietary GI= (sum of GL/ g available carbohydrates)*100; ^3^dietary GL= sum of GL ^4^ Lunch was freshly cooked. Nutritional and GI values refer to raw ingredients;  ^5^Tomato sauce was self-made: canned tomatoes (mashed) (GI=52, GL= 2; mean of green peas, sweet corn, carrots, pumpkin [18], tomato puree (GI=52, GL=1; mean of green peas, sweet corn, carrots, pumpkin [18], vegetable broth, dried herbs, salt, and pepper (all GI=0, GL=0); ^6^ dressing: olive oil, herbs, salt (all GI=0, GL=0); ^7^ total sum deviates from individual data listed due to rounding of decimals; ^8^ 1% accounts for organic acids. ^9^ 2 kcal account for 1g fiber to achieve 100% total energy

**Supplemental Table 3:** Change in anthropometric characteristics of the study population before and after the controlled nutrition trial.

|  | **Before intervention**  **(day 1)**^1^ | **After intervention**  **(day 8)**^2^ |
| --- | --- | --- |
| **Early chronotypes (n=22)** | | |
| BMI, kg/m^2^ | 22.4 (± 2.2) | 22.1 (± 2.1) |
| Waist circumference, m | 0.7 (0.7; 0.8) | 0.7 (0.7; 0.8) |
| Visceral fat mass, L | 0.4 (0.3; 0.6) | 0.5 (0.3; 0.6) |
| Skeletal muscle mass, kg | 23.2 (20.1; 28.4) | 23.1 (20.1; 27.7) |
| **Late chronotypes (n=23)** | | |
| BMI, kg/m^2^ | 22.5 (± 2.6) | 22.3 (± 2.5) |
| Waist circumference, m | 0.8 (0.8; 0.9) | 0.8 (0.8; 0.9) |
| Visceral fat mass, L | 0.7 (0.4; 1.3) | 0.7 (0.4; 1.2) |
| Skeletal muscle mass, kg | 24.9 (21.2; 31.3) | 24.7 (21.1; 31.4) |

Abbreviations: BMI, body mass index. Anthropometric measurements were performed at different daytimes: ^1^ in the afternoon; ^2^ in the morning. Data are frequencies, means ± standard deviation, or medians (Q1, Q3).


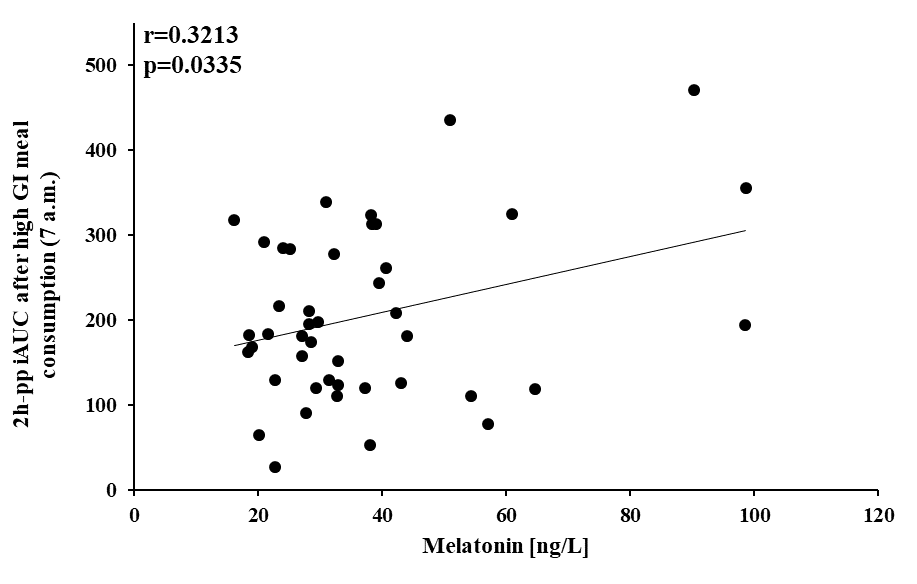


**Supplemental figure 1:** **Association between 2h-pp iAUC after high-GI meal consumption in the morning (7 a.m.) and morning melatonin values (n=44).**
